# Older fusion-surgery age in congenital scoliosis patients is a risk factor for extended length of stay, more estimated blood loss, longer fused segments and higher medical costs: a retrospective study

**DOI:** 10.1186/s12891-021-04650-6

**Published:** 2021-09-12

**Authors:** Xiran Chai, Guanfeng Lin, Shengru Wang, Yang Yang, Zhe Su, You Du, Xiaolin Xu, Xiaohan Ye, Jianxiong Shen, Jianguo Zhang

**Affiliations:** Departments of Orthopaedic Surgery, Peking Union Medical College Hospital (PUMCH), 1st Shuai Fu Yuan Hutong, Dongcheng District, 100730 Beijing, People’s Republic of China

**Keywords:** Congenital scoliosis, Risk factor for fusion surgery, Length of stay, Estimated blood loss, Medical costs

## Abstract

**Background:**

Contradictory opinions about whether early correction and fusion surgeries should be performed for congenital scoliosis (CS) patients at a young age exist. The objectives of this study were to analyze the association between patient characteristics and fusion-surgery outcomes in CS patients treated with spinal correction and fusion surgeries and to report risk factors for extended length of stay (LOS), more estimated blood loss (EBL), longer fused segments and higher medical costs.

**Methods:**

We analyzed data of 1,207 CS inpatients treated with fusion surgeries in our institute from January 2010 - December 2019. All patients underwent spinal X-ray, CT, MRI, echocardiogram and urogenital ultrasound. We analyzed demographic and clinical information and outcome measures, including LOS, EBL, fused segments and medical costs.

**Results:**

Age at fusion (OR = 1.053; *p* < 0.001), musculoskeletal defects (OR = 1.670; *p* = 0.004) and thoracic deformity (OR = 1.519; *p* = 0.03) were risk factors for extended LOS. Age at fusion (OR = 1.117; *p* < 0.001), male sex (OR = 1.813; *p* < 0.001), mixed defects (OR = 1.662; *p* = 0.027) and failure of formation (OR = 1.718; *p* = 0.021) were risk factors for more EBL. Age at fusion (OR = 1.213; *p* < 0.001) was a risk factor for longer fused segments. Age at fusion (OR = 1.091; *p* < 0.001) and thoracic deformity (OR = 1.853; *p* = 0.004) were risk factors for higher medical costs.

**Conclusions:**

We found that older age at fusion in CS patients is a risk factor for extended LOS, more EBL, longer fused segments and higher medical costs with the risk increasing by 5–21 % for each year of age. Other identified risk factors include thoracic deformity for extended LOS; longer fused segments, higher medical costs, and musculoskeletal defects for extended LOS; and CS type (FF and MD) and sex (male) for more EBL.

## Background

Congenital scoliosis (CS) is the lateral curvature of the spine resulting from vertebral deformities that develop during the 4th to 6th weeks of gestation and are present at birth. CS was reported to have an incidence of approximately 1 in every 1,000 live births [1]. Surgery for CS is indicated for cases of documented/potential progressive curvature and/or neurological deficits and remains one of the most challenging surgeries in orthopedics.

Unlike the etiologies of other types of scoliosis, such as idiopathic, neuromuscular and syndromic, the deformity-causing forces of CS are due to only congenital vertebral malformation. Once the congenital vertebral malformation is corrected, most scoliosis will no longer progress. However, if the congenital vertebral malformation is left intact, 50 % of CS displays very rapid progression [2]. The progression of CS depends on the imbalance of growth potential and the growth rate of the spine. The growth rate of the spine is not uniform, and there are periods of accelerated growth, before age 5 and during the adolescence growth spurt [3]. Thus, early congenital deformity correction and spine fusion might be beneficial for patients to prevent scoliosis from progressing. However, there are still contradictory opinions about whether early correction and fusion surgeries should be performed for patients at a younger age. Extended length of stay (LOS), more estimated blood loss (EBL), and long fusion segments are important concerns against early surgeries. However, the association between patient characteristics, including age at surgeries, and LOS, EBL and fusion segments has scarcely been reported.

The objectives of this study were to systematically analyze the association between patient characteristics and surgery outcomes and to report risk factors for extended LOS, more EBL, longer fused segments and higher medical costs.

## Methods

### Patients

We retrospectively searched the inpatient database for cases in which patients were admitted for CS and underwent spine fusion surgeries from January 2010 to December 2019 in our institute using ICD-10 codes (Q67.501, Q67.502). The inclusion criteria were (1) patients diagnosed with CS and (2) patients who underwent primary posterior spine fusion surgery with or without osteotomies. In total, 1,207 inpatients were included in our study. The exclusion criteria were as follows: (1) patients with scoliosis caused by non-congenital factors, such as idiopathic and neuromuscular factors, and (2) patients who underwent non-fusion surgeries or in whom an anterior/anterior-posterior approach was employed.

Demographic information, including sex and age at the initial operation, was directly extracted from the inpatient database. The CS types, namely failure of formation (FF), failure of segmentation (FS) and mixed defects (MD), were diagnosed from the patients’ X-ray and spine CT [20,21]. Echocardiogram; urogenital ultrasound; spine imaging; including MRI, X-ray and CT; and systemic physical examination were undertaken in all patients to screen and diagnose associated anomalies. Primary outcomes, including the length of stay, estimated blood loss, fusion segments (two vertebral bodies and an intervertebral disc were defined as two levels or one segment), medical costs and complications, were extracted from medical records and analyzed.

### Statistical analysis

All statistical analyses were performed using SPSS Statistics Version 23 (IBM, Armonk, New York). An independent Student’s t test was used to analyze continuous data with a normal distribution. A nonparametric test was used to analyze continuous data with a non-normal distribution. The chi-square test was used to analyze enumerated data. In the univariate analysis, factors with a *P*-value < 0.1 were included as potential risk factors in multiple logistic regression analysis to determine significant independent risk factors. A *P*-value < 0.05 was considered statistically significant in multivariate analysis. All graphs were plotted by GraphPad Prism 8 (Version 8.4.0, GraphPad Software, LLC.).

## Results

From January 2010 to December 2019, a total of 1,207 inpatients who underwent instrumented fusion surgery were included in this study. The average age at the time of fusion surgery was 12.7 ± 8.2 years old and is significantly lower in male than in female (11.51 vs. 13.72, *p* < 0.001). 564 patients (46.7 %) were male, while 643 patients (53.3 %) were female. 50 % of patients (*n* = 604) had failure of formation, 18.5 % of patients (*n* = 223) had failure of segmentation and the remaining 31.5 % of patients (*n* = 380) had mixed defects. Regarding vertebral anomaly location, cervical, thoracic and lumbar vertebral anomalies were found in 67 patients (5.6 %), 930 patients (77.1 %) and 423 patients (35.1 %), respectively (Table 1).

**Table 1:** Basic information

Demographic information	
Sample size	1,207
Sex (male/female)	564/643
Age at fusion (year)	12.69 ± 8.19
Age at fusion (male)	11.51 ± 7.82
Age at fusion (female)	13.72 ± 8.38 (*p* < 0.001)
Deformity information	
CS type (FF/FS/MD)	604/223/380 (50.04 %/18.48 %/31.48 %)
Cervical involved	5.55 % (*n* = 67)
Thoracic involved	77.05 % (*n* = 930)
Lumbar involved	35.05 % (*n* = 423)
Comorbidity information	
Intraspinal	28.58 % (*n* = 345)
Musculoskeletal	15.49 % (*n* = 187)
Cardiac	13.67 % (n = 165)
Gastrointestinal	3.65 % (*n* = 44)
Urogenital	5.80 % (*n* = 70)
Outcome information	
Length of stay > 14 days	23.86 % (*n* = 288)
Estimated blood loss > 800 ml	20.21 % (*n* = 244)
Fusion segments > 8 levels	48.63 % (*n* = 587)
Medical costs > 20,000 USD	22.04 % (*n* = 266)

With regard to comorbidity information, the incidences of intraspinal, musculoskeletal, cardiac, gastrointestinal and urogenital defects were 28.6 % (*n* = 345), 15.5 % (*n* = 187), 13.7 % (*n* = 165), 3.6 % (*n* = 44) and 5.8 % (*n* = 70), respectively. Twenty-three point nine percent (*n* = 288) of patients had an extended length of stay (defined as LOS > 14 days), 20.2 % (*n* = 244) of patients had more estimated blood loss (defined as EBL > 800 ml), 48.6 % (*n* = 587) of patients had more than 8 levels fused and 22.0 % (*n* = 266) of patients had medical costs greater than 20,000 USD (Table 1).

**Fig. 1:**
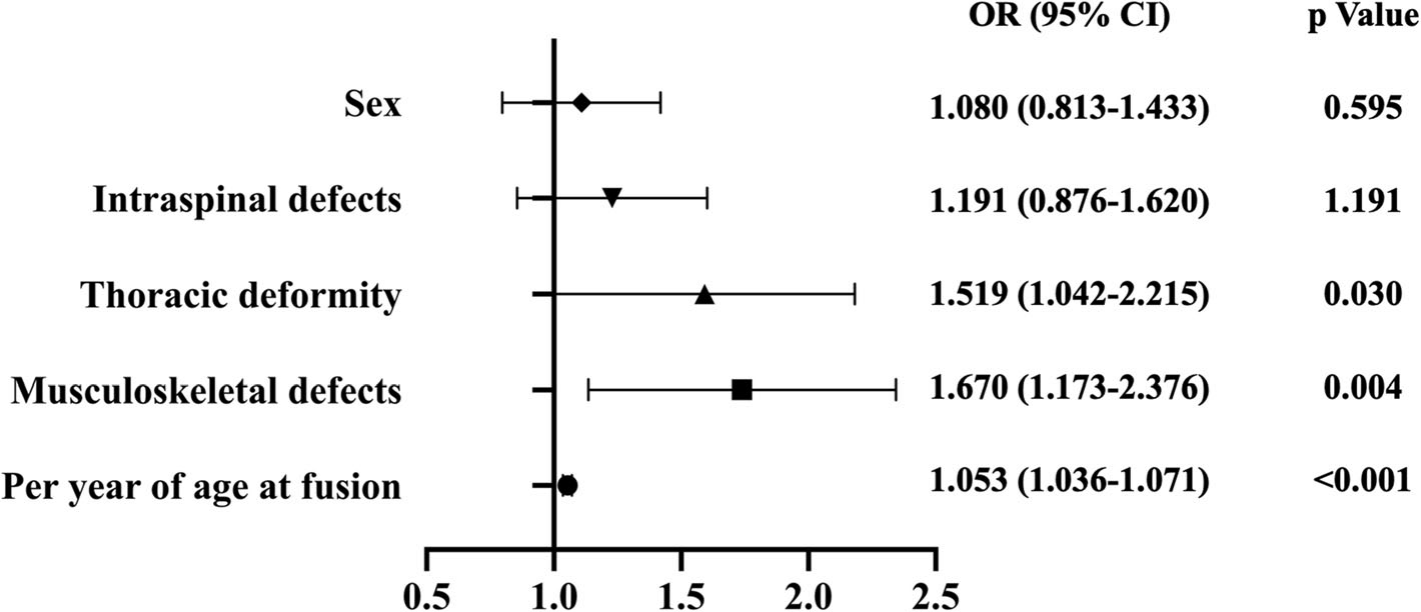
Odds ratio and *p* value of multivariate analysis of extended length of stay (length of stay > 16 days)

To avoid missing potential risk factors, *P*-values < 0.1 were considered statistically significant in the univariate analysis. After the univariate analysis, significant risk factors for extended LOS included sex, age at operation, CS type, thoracic deformity, intraspinal defects and musculoskeletal defects (Table 2). Among risk factors with *P*-value < 0.1 in the univariate analyses, age at operation (OR = 1.053 [1.036–1.071]; *p* < 0.001), musculoskeletal defects (OR = 1.670 [1.173–2.376]; *p* = 0.004) and thoracic deformity (OR = 1.519 [1.042–2.215]; *p* = 0.03) were found to be independent risk factors for extended LOS in multivariate analyses (Fig. 1).

**Table 2:** Univariate analysis and multivariate analysis of LOS risk factors

Parameters	LOS > 16 days	LOS ≤ 16 days	*p*
Univariate analysis			
Sex (male/female)	122/166	442/477	0.089
Age at fusion	15.70 ± 10.15	11.75 ± 7.23	< 0.001
CS type (FF/FS/MD)	41.3 %/19.1 %/39.6 %	52.8 %/18.3 %/28.9 %	0.001
Cervical deformity	7.3 %	5.0 %	0.139
Thoracic deformity	84.4 %	74.8 %	0.001
Lumbar deformity	31.3 %	36.2 %	0.122
Intraspinal defects	35.8 %	26.3 %	0.002
Musculoskeletal defects	22.2 %	13.4 %	< 0.001
Cardiac defects	15.3 %	13.2 %	0.363
Gastrointestinal defects	2.8 %	3.8 %	0.368
Urogenital defects	7.3 %	5.3 %	0.214
Parameters	*p*	Odds ratio	95 % CI
Multivariate analysis			
Age at fusion	< 0.001	1.053	1.036–1.071
Musculoskeletal defects	0.004	1.670	1.173–2.376
Thoracic deformity	0.030	1.519	1.042–2.215
Intraspinal defects	0.264	1.191	0.876–1.620
Sex	0.595	1.080	0.813–1.433

**Fig. 2:**
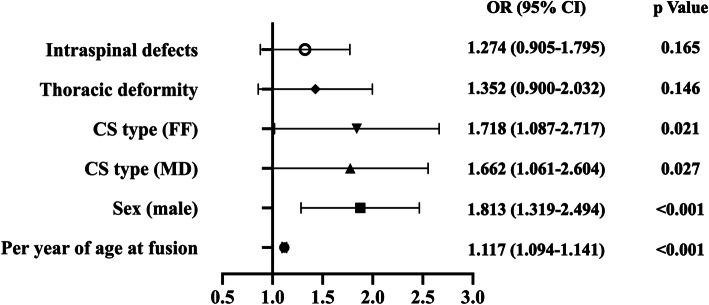
Odds ratio and *p* value of multivariate analysis of estimated blood loss during fusion surgery (estimated blood loss > 800ml)

In the analysis of EBL risk factors, sex, age at operation, CS type, thoracic deformity and intraspinal defects were recognized as potential risk factors after univariate analysis. After multivariate analysis, age at operation (OR = 1.117 [1.094–1.141]; *p* < 0.001), male (OR = 1.813 [1.319–2.494]; *p* < 0.001), CS types of mixed defects (OR = 1.662 [1.061–2.604]; *p* = 0.027) and failure of formation (OR = 1.718 [1.087–2.717]; *p* = 0.021) were found to be independent risk factors for estimated blood loss greater than 800 ml (Fig. 2) (Table 3).

**Table 3:** Univariate analysis and multivariate analysis of EBL risk factors

Parameters	EBL > 800 ml	EBL ≤ 800 ml	*p*
Univariate analysis			
Sex (male/female)	128/116	436/527	0.045
Age at fusion	18.44 ± 8.56	11.23 ± 7.42	< 0.001
CS type (FF/FS/MD)	45.9 %/16.8 %/37.3 %	51.1 %/18.9 %/30.0 %	0.091
Cervical deformity	7.0 %	5.2 %	0.279
Thoracic deformity	82.0 %	75.8 %	0.041
Lumbar deformity	36.5 %	34.7 %	0.600
Intraspinal defects	34.8 %	27.0 %	0.016
Musculoskeletal defects	17.6 %	15.0 %	0.303
Cardiac defects	12.7 %	13.9 %	0.623
Gastrointestinal defects	3.7 %	3.6 %	0.968
Urogenital defects	6.6 %	5.6 %	0.571
Parameters	*p*	Odds ratio	95 % CI
Multivariate analysis			
Age at fusion	< 0.001	1.117	1.094–1.141
Sex (male)	< 0.001	1.813	1.319–2.494
CS type	0.047	-	-
FS	-	1 (Reference)	
MD	0.027	1.662	1.061–2.604
FF	0.021	1.718	1.087–2.717
Thoracic deformity	0.146	1.352	0.900-2.032
Intraspinal defects	0.165	1.274	0.905–1.795

**Fig. 3:**
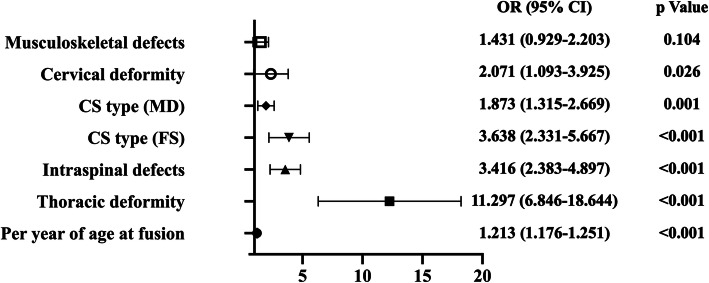
Odds ratio and *p* value of multivariate analysis of long-segment fusion. Long-segment fusion was defined as more than 8 segments fused. One segment was defined as two adjacent vertebrae and the intervertebral disc between them

Regarding the analysis of long-segment fusion, age at operation (OR = 1.213 [1.176–1.251]; *p* < 0.001), thoracic deformity (OR = 11.297 [6.846–18.644]; *p* < 0.001), intraspinal defects (OR = 3.416 [2.383–4.897]; *p* < 0.001), CS types of failure of segmentation (OR = 3.638 [2.331–5.667]; *p* < 0.001), mixed defects (OR = 1.873 [1.315–2.669]; *p* = 0.001) and cervical deformity (OR = 2.071 [1.093–3.925]; *p* = 0.026) were identified as independent risk factors for more than 8 levels fused (Fig. 3) (Table 4).

**Table 4:** Univariate analysis and multivariate analysis of long-segment fusion risk factors

Parameters	Fused segments > 8	Fused segments ≤ 8	*p*
Univariate analysis			
Sex (male/female)	225/362	339/281	< 0.001
Age at fusion	16.61 ± 7.80	8.98 ± 6.69	< 0.001
CS type (FF/FS/MD)	29.1 %/30.0 %/40.9 %	69.8 %/7.6 %/22.6 %	< 0.001
Cervical deformity	7.3 %	3.9 %	0.009
Thoracic deformity	93.4 %	61.6 %	< 0.001
Lumbar deformity	20.3 %	49.0 %	< 0.001
Intraspinal defects	45.3 %	12.7 %	< 0.001
Musculoskeletal defects	20.8 %	10.5 %	< 0.001
Cardiac defects	15.0 %	12.0 %	0.194
Gastrointestinal defects	2.4 %	4.8 %	0.023
Urogenital defects	5.8 %	5.8 %	0.992
Parameters	*p*	Odds ratio	95 % CI
Multivariate analysis			
Age at fusion	< 0.001	1.213	1.176–1.251
Thoracic deformity	< 0.001	11.297	6.846–18.644
Intraspinal defects	< 0.001	3.416	2.383–4.897
CS type	< 0.001	-	-
FF	-	1 (Reference)	
FS	< 0.001	3.638	2.331–5.667
MD	0.001	1.873	1.315–2.669
Cervical deformity	0.026	2.071	1.093–3.925
Musculoskeletal defects	0.104	1.431	0.929–2.203

**Fig. 4:**
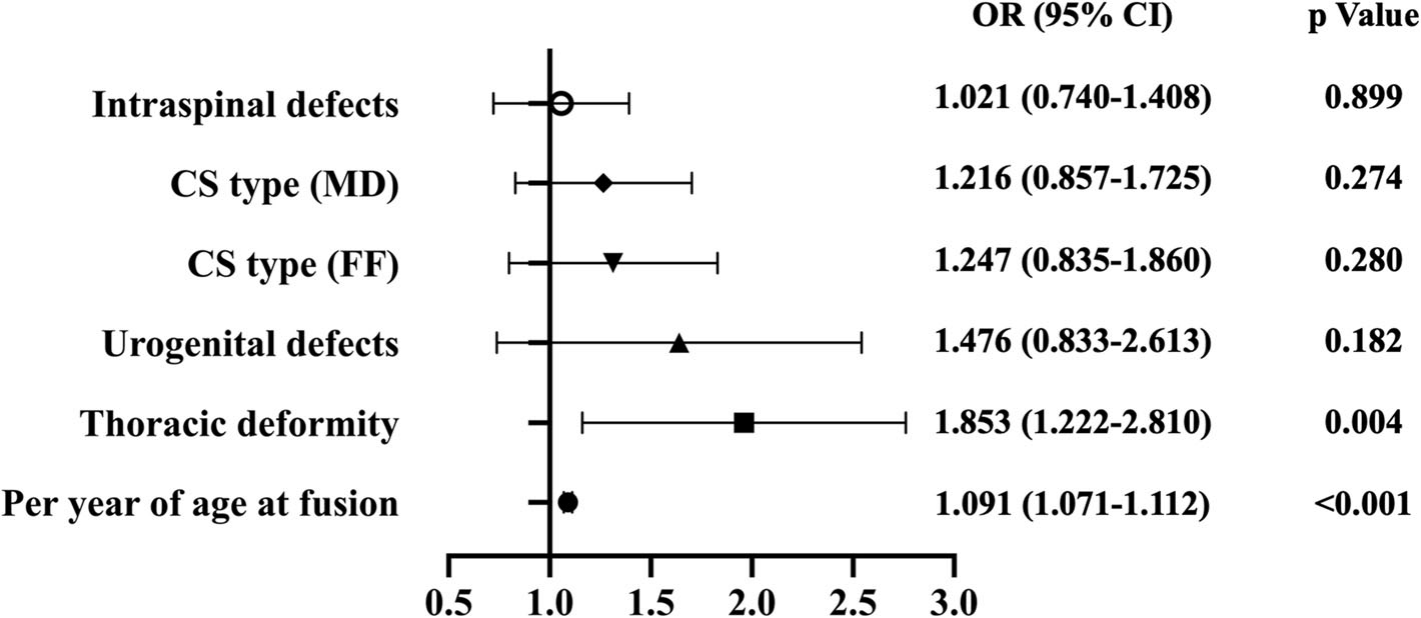
Odds ratio and *p* value of multivariate analysis of higher medical costs (medical costs > 20,000 USD)

When analyzing risk factors for medical costs, we found that patients with medical costs greater than 20,000 USD had older age at operation (*p* < 0.001), different CS type distribution (*p* < 0.001), more patients with thoracic deformity (*p* < 0.001) and more patients with intraspinal (*p* = 0.009) and urogenital defects (*p* = 0.024) in univariate analysis. Although lumbar deformity had a *P*-value < 0.1 in univariate analysis, we excluded it from multivariate analysis, as it appeared to be a protective factor for higher medical costs. After multivariate analysis, age at operation (OR = 1.091 [1.071–1.112]; *p* < 0.001) and thoracic deformity (OR = 1.853 [1.222–2.810]; *p* = 0.004) were identified as independent risk factors for medical costs greater than 20,000 USD (Fig. 4) (Table 5).

**Table 5:** Univariate analysis and multivariate analysis of medical costs risk factors

Parameters	Medical costs > 20k USD	Medical costs ≤ 20k USD	*p*
Univariate analysis			
Sex (male/female)	126/140	438/503	0.812
Age at fusion	17.67 ± 9.10	11.28 ± 7.33	< 0.001
CS type (FF/FS/MD)	38.0 %/24.4 %/37.6 %	53.5 %/16.8 %/29.8 %	< 0.001
Cervical deformity	5.6 %	5.5 %	0.943
Thoracic deformity	86.1 %	74.5 %	< 0.001
Lumbar deformity	28.9 %	36.8 %	0.018
Intraspinal defects	35.0 %	26.8 %	0.009
Musculoskeletal defects	16.2 %	15.3 %	0.731
Cardiac defects	15.0 %	13.3 %	0.462
Gastrointestinal defects	2.3 %	4.0 %	0.171
Urogenital defects	8.6 %	5.0 %	0.024
Parameters	*p*	Odds ratio	95 % CI
Multivariate analysis			
Age at fusion	< 0.001	1.091	1.071–1.112
Thoracic deformity	0.004	1.853	1.222–2.810
Urogenital defects	0.182	1.476	0.833–2.613
CS type	0.448	-	-
FF	-	1 (Reference)	
FS	0.280	1.247	0.835–1.860
MD	0.274	1.216	0.857–1.725
Intraspinal defects	0.899	1.021	0.740–1.408

The total osteotomy rate in our CS cohort is 56.7 % (*n* = 684) (Table 6). After analyzing the correlation between osteotomy rate and LOS, EBL and medical costs, we found that patients who underwent osteotomy had lower incidences of LOS > 14 days (37.6 % vs. 42.6 %, *p* = 0.075) and medical costs > 20,000USD (20.0 % vs. 24.7 %, *p* = 0.059) and higher incidences of EBL > 800ml (22.4 % vs. 17.4 %, *p* = 0.036).

**Table 6:** Osteotomy information

Osteotomy information		
Total osteotomy rate	56.7 % (*n* = 684)	
Correlation between osteotomy and LOS		
LOS > 14 days in osteotomy group	37.6 % (257/684)	*p* = 0.075
LOS > 14 days in non-osteotomy group	42.6 % (223/523)	
Correlation between osteotomy and EBL		
EBL > 800ml in osteotomy group	22.4 % (153/684)	*p* = 0.036*
EBL > 800ml in non-osteotomy group	17.4 % (91/523)	
Correlation between osteotomy and medical cost		
Cost > 20,000USD in osteotomy group	20.0 % (137/684)	*p* = 0.059
Cost > 20,000USD in non-osteotomy group	24.7 % (129/523)	

*p < 0.05

As for the analysis of complication information (Table 7), we found that the total complication rate in our study is 13.2 % (*n* = 159) and complication rate in patients of 0–3 years old and in patients of more than 3 years old showed no statistically significance (8.7 % vs. 13.8 %, *p* = 0.109). Rates for specific complications were 2.0 % for implantation-related complications, 2.0 % for adding-on and decompensation, 1.3 % for incision-related complications, 1.1 % for infections, 3.3 % for pleural effusion and hemothorax, 2.0 % for neurologic complications and 0.4 % for CSF leakage.

**Table 7:** Complication information

Complication information		
Total complications	13.2 % (*n* = 159)	
Complications in 0–3 year-old patients	8.7 % (12/138)	*p* = 0.109
Complications in > 3 year-old patients	13.8 % (147/1069)	
Implantation-related complications	2.0 % (*n* = 23)	
Adding-on and decompensation	2.0 % (*n* = 23)	
Incision-related complications	1.3 % (*n* = 16)	
Infections	1.1 % (*n* = 13)	
Pleural effusion, hemothorax	3.3 % (*n* = 40)	
Neurologic complications	2.0 % (*n* = 24)	
CSF leakage	0.4 % (*n* = 5)	

## Discussion

The spine undergoes a bimodal growth spurt: once during the first 3 to 5 years of life and again during puberty [3–5]. Thus, congenital vertebral malformations (CVMs) in patients at early ages would result in rapid progression of scoliosis during these two growth spurts and lead to severe prognosis until the skeleton fully matures. As a result, some surgeons recommended that early surgery and CVM correction should be performed to prevent scoliosis from progressing. However, early surgery, especially long-segment fusion, might increase the risk of damaged development of the lung, thoracic cage and vertebrae. These controversies have lasted for decades. There is still a lack of studies in large-scale samples reporting effect of fusion age on surgical safety and outcomes.

The most prevalent concern about early fusion is damaged pulmonary function. CS patients treated with early spinal fusion have lower forced vital capacity, forced expiratory volume, vital capacity and total lung capacity than healthy children, and the impairment of pulmonary function is more severe in patients with thoracic fusions [6]. Early spinal fusion and longer fused segments are also associated with more severe damage to vital capacity [7, 8]. However, these studies all compared patients with severe congenital deformities that require early surgical correction to healthy people or those with mild congenital deformities that can be treated with conservative treatment. Patients with severe congenital deformities would have lower pulmonary function by nature; thus, we could not conclude that their more severe lung function damage is due to early spinal fusion or compression caused by severe deformities from these studies. Complications, such as the crankshaft phenomenon, are another problem. The crankshaft phenomenon was reported to be positively associated with earlier spinal fusions [9]. In short, there are still concerns, such as pulmonary function damage and other complications, such as the crankshaft phenomenon, about the effects of fusion surgeries on immature spines among spinal surgeons.

Another concern is that pedicle screws have to pass through the neurocentral cartilage (NCC), which is responsible for the growth of the pedicles and the posterior vertebral body, and the NCC begins to fuse at 3 to 5 years of age [10]. Thus, there is concern that pedicle screw instrumentation in patients younger than 5 years of age may affect the development of the vertebral body and spinal canal. Until now, there has been no evidence of iatrogenic vertebral dysplasia or spinal stenosis due to pedicle screws. Michael et al. [11] studied the use of pedicle screws in pediatric patients younger than 2 years old and concluded that pedicle screw fixation can be safely performed without more complications related to pedicle screw insertion or negative effects on vertebral growth. Several later studies corroborated that the growth rates of the vertebral body and spinal canal were not affected by pedicle screw instrumentation at a young age [12–14]. In terms of the effect on the growing spine, pedicle screw instrumentation is widely accepted to be safe in infants.

By directly analyzing the interrelationship between age at fusion and patient outcomes, Wu et al. [15] found that older age was an independent risk factor for the development of pulmonary complications after posterior spinal instrumentation and fusion in CS patients. According to our data in this study, we also found that older age at surgery is a risk factor for extended LOS, more EBL, longer fused segments and higher medical costs with the risk increasing by 5–21 % for each year increase in patient age at the time of fusion surgery (Figs. 1, 2, 3 and 4). This result demonstrated that it is necessary and reasonable to perform corrective surgeries as early as possible to reduce medical costs both for hospitals and for patients and to preserve motive segments. Moreover, we further analysis complications in different ages and found that surgery in earlier age did not increase complication rates (Table 7). The analysis of complications revealed that fusion surgery in patients under 3 years old would not lead to higher complication rate, further supporting the safety of early fusion surgery. However, the appropriate age for surgery must be very carefully selected. In our CS series, patients with simple hemivertebrae were treated with posterior hemivertebra resection and short-segment fusion using pedicle screws no earlier than 1.5 years old, as previous studies have indicated. Good correction rates were obtained in these patients, with few complications. Based on our clinical experience, we also found that young children treated with osteotomy and short-segment fusion grew to a height similar to that of their healthy peers after skeletal maturity. For other patients, if the congenital deformity was limited to a small region, or if the main cause of spinal deformity was limited, we adopted a strategy of early posterior osteotomy and short-segment fusion to eliminate the cause of spinal deformity and correct local scoliosis. The outcomes of these patients were satisfactory. However, we also found that osteotomy surgery was correlated with significantly higher incidences of EBL > 800ml (Table 6). Therefore, it is better to perform comprehensive consideration preoperatively and conduct osteotomy surgery by experienced surgeons.

Theoretically speaking, early resection of congenital vertebral deformities and correction of scoliosis are beneficial to prevent the development of severe local deformities and secondary curves. Once secondary curves become structured, the extension of instrumented fused segments to comprise these curves is unavoidable. Earlier surgeries lead to milder deformities, shorter fusion segments and better outcomes. This strategy of early surgery is effective, particularly for CS patients with a single hemivertebra, in which case the hemivertebra is the only cause of deformity. As long as hemivertebrae are completely resected and local scoliosis is appropriately corrected, there was no risk of further progression and the formation of structured secondary curves [16]. This procedure also enables surgeons to preserve the motive segments and allows for normal growth in the unaffected parts of the spine.

Chang et al. [12] demonstrated the safety and effectiveness of posterior hemivertebra resection and short-segment fusion using segmental pedicle screw fixation in CS patients younger than 10 years at the time of the surgery with a mean follow-up of 11.4 years. No crankshaft phenomenon, spinal stenosis or major complications related to the pedicle screws were found during the follow-up period. As very little growth potential of the spinal canal remains after 3 years of age [17], the authors suggested that pedicle screw instrumentation later than 2 years of age would prevent the occurrence of spinal stenosis. They also concluded that patients treated with earlier hemivertebra excision and posterior short-segment fusion with pedicle screws before the age of 6 years had better outcomes than those aged 7 to 10 years. In another CS series, Crostelli et al. [18] performed posterior hemivertebra resection and short-segment fixation using pedicle screws in 15 patients aged 18 months to 9.5 years, concluding that this procedure led to significant advances in congenital deformity control and a low rate of neurological complications for patients as young as 18 months old. This result is in accordance with those of other independent studies [11, 19–24]. The appropriate age at surgery is generally recommended to be 1.5 to 2 years old. Except for patients with simple hemivertebrae, Ruf et al. [24] also found that posterior osteotomy with short-segment fusion using transpedicular instrumentation had good efficacy and safety to treat young patients with bar formation.

We also found that thoracic deformity was a risk factor for extended LOS, longer fused segments and higher medical costs, with an odds ratio from 1.67 to 11.30 (Figs. 1, 3 and 4). In our previous clinical practice, we found that thoracic deformities are associated with more failure of segmentation and mixed defects and that the curvatures are usually more severe than cervical and lumbar deformities. Moreover, thoracic deformities were found to have higher incidences of musculoskeletal [25] and intraspinal defects [26, 27]. Thus, because of the restriction of the thoracic cage and the negative effect of musculoskeletal defects, such as parallel ribs, patients with thoracic vertebral malformations tend to have more complex cases and more severe deformities, requiring more challenging surgeries with higher grade osteotomies and longer fused segments. This might help to explain the association between thoracic deformity and extended LOS, longer fused segments and higher medical costs found in our study.

Other identified risk factors include musculoskeletal defects for extended LOS (Fig. 1) and CS type (FF and MD) and sex (male) for more EBL (Fig. 2). Patients with failure of formation and mixed defects usually require surgeons to perform osteotomies to resect hemivertebrae or other congenital malformations and thus risk more perioperative blood loss. These several risk factors should be taken into consideration by surgeons in their preoperative planning and patient informing.

Generally, the strategy of early surgery is effective and safe for CS patients with local congenital vertebral malformations to minimize deformities at surgery, fusion segments, surgical trauma and medical costs. In our clinical practice, these patients, who underwent early osteotomy and short-segment fusion, were observed to have growth potential that was similar to that of their healthy peers. Thus, for CS patients with local congenital vertebral malformation and limited scoliosis, we recommended that spinal surgery be performed as early as 1.5 to 2 years of age to eliminate most forces that lead to the progression of spinal deformity. Otherwise, due to its natural history, deformities will become more severe as CS patients age and require greater surgeries and longer fusion segments, which will lead to more EBL, higher costs and longer LOS.

In summary, our study has two major strengths. First, to our knowledge, this is one of the largest single-center CS cohorts to date. Second, we are the first to comprehensively analyze the association between demographic and clinical information and outcome measures in a large CS cohort treated in a single center. However, as this is a retrospective study of data extracted from medical records, the quality and reliability of the results of this study were largely influenced by the recorders, imaging interpreters and medical record quality.

## Conclusions

We found that older age at surgery was a risk factor for extended LOS, more EBL, longer fused segments and higher medical costs, with the risk increasing by 5–21 % for each year increase in patient age at the time of fusion surgery. Thoracic deformity is a risk factor for extended LOS, longer fused segments and higher medical costs. Other identified risk factors include musculoskeletal defects for extended LOS, CS type (FF and MD) and sex (male) for more EBL.

## Data Availability

The data set in this study is partially included in another undisclosed and uncompleted study, authors of which didn’t approve to share their patients’ clinical data for both public and peer review purposes.

## Abbreviations

CS: Congenital scoliosis
FF: Failure of formation
FS: Failure of segmentation
MD: Mixed defects
LOS: Length of stay
EBL: Estimated blood loss
